# The Heck Reaction in Ionic Liquids: Progress and Challenges

**DOI:** 10.3390/molecules15042211

**Published:** 2010-03-30

**Authors:** Fabio Bellina, Cinzia Chiappe

**Affiliations:** Dipartimento di Chimica e Chimica Industriale, via Risorgimento 35, 56126 Pisa, Italy

**Keywords:** ionic liquids, heck reaction, Pd catalyst, selectivity

## Abstract

As the interest for environmental increases and environmental laws become more stringent, the need to replace existing processes with new more sustainable technologies becomes a primary objective. The use of ionic liquids to replace organic solvents in metal catalyzed reactions has recently gained much attention and great progress has been accomplished in this area in the last years. This paper reviews the recent developments in the application of ionic liquids and related systems (supported ionic liquids, ionic polymers, and so on) in the Heck reaction. Merits and achievements of ionic liquids were analyzed and discussed considering the possibility of increasing the effectiveness of industrial processes.

## 1. Introduction

Over the past decade, ionic liquids (ILs), salts having melting points lower than 100 °C, have become one of the fastest growing research areas. The application of these remarkable salts as solvents in organic reactions and catalytic processes has been extensively investigated and reviewed [1,2,3,4,5,6,7,8,9]. The term “ionic liquid” identifies a really large class of compounds, constituted exclusively by ions, which are liquid at/or near room temperature and present unique physicochemical properties. As first, the ionic nature determines the lack of a measurable vapor pressure and this feature, associated with a high thermal and chemical stability, makes these compounds intrinsically excellent candidates for industrial applications compared to common organic solvents. This is particularly true considering that ILs can nicely complement, and even sometimes work better, than organic solvents in a number of industrial processes [1,2,3]. 

The Heck reaction represents not only the most extensively investigated Pd-catalyzed process in ILs, but, the related studies practically cover all the development of ILs chemistry up today; they start with the use of onium salts as reaction media, continue with the pyridinium and imidazolium ones to arrive, more recently, to the supported ILs passing through the so-called task-specific ionic liquids, TSILs. This intense activity in this field is probably due to the fact that the Heck coupling is an extremely popular reaction, of industrial interest, which is very versatile but presents a number of well-known problems and it is generally considered the classical example of palladium catalyzed carbon-carbon bond formation process. Heck arylation of electron-deficient olefins have been investigated in ILs mainly to verify the effect of medium on the catalytic system; activity, selectivity and stability of the catalyst under ligand free-conditions, as well as the possibility of catalyst and solvent recovery and recycle have been tested under different conditions. In the case of arylation of electron-rich olefins also the effect on the medium on regioselectivity has been investigated, whereas the possibility of use alternative arylating agents has been rarely evaluated; many of the reported data is related to the reaction of aryl halides (iodides and bromides) with styrenes and acrylates. 

## 2. Heck Reaction in Common ILs

Simple palladium salts such as PdCl_2_ or Pd(OAc)_2_ in the absence of stabilizing phosphine ligands have been widely used in the Heck reaction in ILs [10,11,12,13,14], and preparatively significant examples have been performed. 

**Scheme 1:**
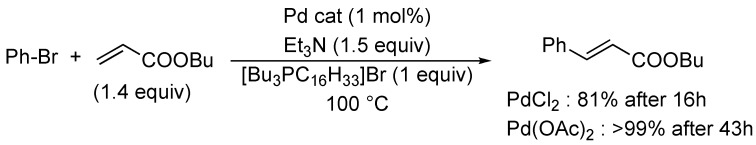
Heck reaction without additive ligands in [C_16_PBu_3_]Br.

In 1984, in a pioneering work Jeffery reported the palladium catalyzed vinylation of organic iodides under solid liquids phase transfer conditions, around room temperature, using tetrabutylammonium chloride [15]. However, the first well-documented application of ILs in the Heck reaction, that is also the first palladium-catalyzed reaction performed in ILs, was reported in 1996 by Kauffmann and co-workers [16]. In particular, they surprisingly found that Pd(OAc)_2_ and PdCl_2_ are both appropriate catalyst precursors for Heck reactions without additive ligands (Scheme 1) in [C_16_PBu_3_]Br. 

The addition of 1.5 equiv. of NaOAc when Pd(OAc)_2_ was used as the catalyst precursor, improved the rate of the coupling, but a concomitant decrease of the selectivity was observed, leading to the formation of 5% of the (*Z*)-isomer. The precipitation of Pd clusters slowly started after some hours when PdCl_2_ was used, but with Pd(OAc)_2_ no precipitation was observed. In this last case the catalyst remained in the melt even at complete conversion of the aryl halide, and after the recover of the product by distillation it resulted active in the following two runs. The authors explained these results by supposing that the phosphonium salt exerted an efficient stabilizing effect on the Pd(0) species obtained in situ by reduction of the Pd(II) catalyst precursors. The results reported by the group of Kauffmann attracted the attention of several research groups on the important challenge of developing new protocols for a ligand-free Heck reaction. In fact, ligand-free conditions in metal-mediated reactions should be always preferred, especially on large scale productions, not only for saving the cost of the ligand but also because the purifications are simplified if no ligand is present [17].

**Scheme 2:**
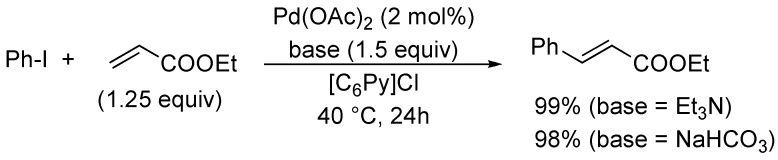
Heck reaction in [C_6_Py]Cl.

In 1999 the Earle group reported the use of hexylpyridium chloride, [C_6_Py]Cl, as solvent in the Heck reaction of iodobenzene with ethyl acrylate using Pd(OAc)_2_ as the catalyst precursor and Et_3_N or NaHCO_3_ as the base (Scheme 2) [18].

Interestingly, the use of the corresponding salts containing the non-coordinating anions hexafluorophosphate and tetrafluoroborate required longer reaction times and higher temperature to go to completion (Table 1).

In contrast to the chloride-based systems, the addition of Ph_3_P as ligand also promoted the reaction when an imidazolium-based IL, [bmim][PF_6_], was used. The authors explained the experimental results, and in particular the hindering on the efficiency of the reaction of a phosphine, suggesting that in a chloride- (or halide-) reach environment the Heck reaction involves a Pd(II)/Pd(IV) redox cycle, whereas in the case where the phosphine ligand is present a Pd(0)/Pd(II) catalyst is operative. However, today a Pd(II)/Pd(IV) mechanism for the Heck reaction is no more accepted. On the contrary, direct NMR measurements and a great deal of experimental data indicate that the Heck reaction proceeds *via* a Pd(0)/Pd(II) catalytic cycle [19]. It is then plausible that the chloride anion contributes to the dissolution of Pd(0) species which come from the reduction of the Pd(II) precatalyst better than BF_4_^-^ or PF_6_^-^. 

Related to this work, it must be remarked that benzoic anhydride, in the absence of a base, has been used as a source of the aryl moiety, although higher temperatures and longer times were required to obtain high conversions. The use of hydrophobic ILs gave the possibility to separate products and salt by-products from the catalysts immobilized in IL (for example, in [bmim][PF_6_]), simply by extraction with ethyl ether and water, respectively [18].

Starting from the results reported by Kauffmann, and taking into account the observations made by Jeffery on the activation performed by salt additives and on the ability of stabilizing the catalytically active Pd species [20], Herrmann and co-workers reported in 1999 their results on ligandless Heck reaction performed in [NBu_4_]Br [21]. As illustrated in Table 2, where the results of these studies have been summarized, PdCl_2_ resulted highly active in promoting the reaction of olefins with iodobenzene (TON = 10000, entry 1), bromoarenes (entries 2-9) and also activated chloroarenes (entries 10–12). However, this catalyst precursor is not capable of coupling 4-chloroanisole efficiently (entry 13).

**Scheme 3:**
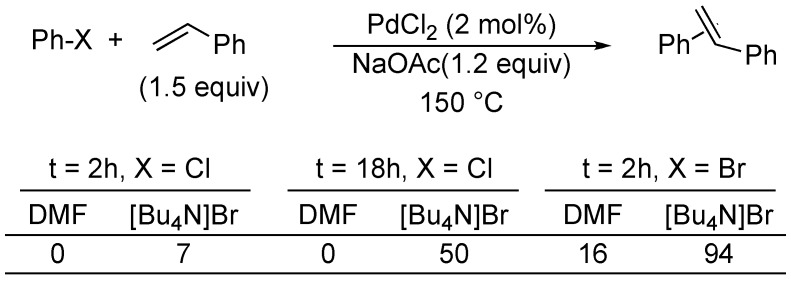
Ligand-free Heck arylation in [Bu_4_N]Br and DMF.

It is noteworthy that PdCl_2_ displayed an increased reactivity in [Bu_4_N]Br when compared with DMF, a “classic” solvent for Heck reactions (Scheme 3).

The unusual long-term thermal stability of the catalyst in [Bu_4_N]Br most probably accounts to the significant differences observed when the reaction was performed at 150 °C. The catalyst decomposes later in the ammonium salt - if it does at all - than it does in DMF under the same conditions. Indeed, the catalyst seems to be more activated; in fact, PdCl_2_ runs even about five time faster in [Bu_4_N]Br than in DMF.

The catalyst and the rather expensive solvent could also be recycled. In fact, in the reaction of bromobenzene and styrene using 1 mol % PdCl_2_ and NaOAc as the base the authors were able to recycle the system several times, nevertheless heavy Pd black formation was observed already during the first run, simply distilling off the reactants and the products *in vacuo*. It was also possible to filter off the NaBr and the Pd black precipitate after 8 recycling runs by dissolving the mixture in acetone. After evaporation of the acetone the filtrate can be used again and stilbene was obtained in 60% yield [22].

The high activity displayed by PdCl_2_ in [Bu_4_N]Br was ultimately attributed by the authors to the thermal reduction of the Pd(II) precatalyst to extremely active colloids. However, it is noteworthy that neither Pd_2_(dba)_3_ nor preformed Pd colloids resulted to actively promote the Heck reaction in [Bu_4_N]Br, confirming that active species have to form *in situ* by reduction. The same ammonium salt, in a mixture with [Bu_4_N]OAc, was employed three years later by Cacchi and co-workers in an efficient stereoselective synthesis of (*E*)- and (*Z*)-3,3-diarylacrylates [23].

**Scheme 4:**
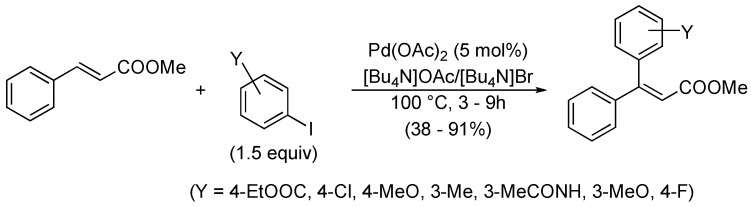
Heck arylation in [Bu_4_N]OAc/[Bu_4_N]Br.

In particular, the Pd-catalyzed reaction of neutral, slightly electron-rich and slightly electron-poor aryl iodides with methyl cinnamate in a molten [Bu_4_N]OAc/[Bu_4_N]Br (2:1.5) mixture provided with high stereoselectivity (*E*)-3,3-diarylacrylates (*E*/*Z* = 98/2 to > 99/1) (Scheme 4). Phosphine-free Pd(OAc)_2_ was employed as the catalyst precursor.

**Scheme 5:**
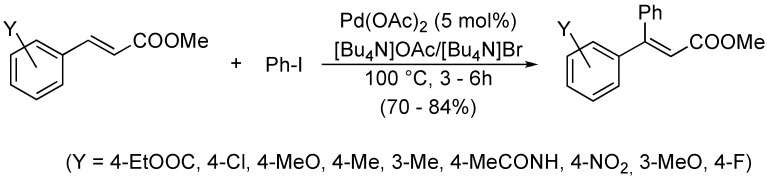
Heck arylation of methyl 3-arylacrylates with iodobenzene.

Moreover, the reaction of a variety of methyl 3-arylacrylates with iodobenzene under the same reaction conditions afforded selectively the corresponding (*Z*)-isomers (*E*/*Z* = 4/96 to <1/>99) (Scheme 5). It was also observed that the catalyst system might be recycled without any loss of activity.

The authors, in order to justify the observed elevated stereoselectivity, attributed to the acetate anion a key role; in particular, they supposed that this anion could favour the irreversible displacement of Pd from σ−Pd adducts thus suppressing any possible isomerization mechanism. They also attributed the high reactivity of Pd(OAc)_2_ in the used molten salt mixture to the formation of palladium nanoparticles stabilized by the ammonium salts.

**Scheme 6:**
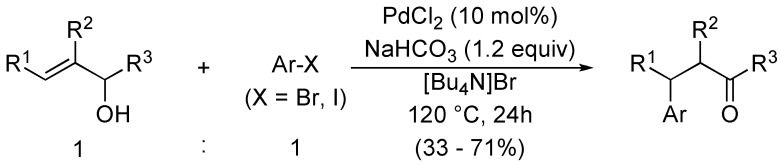
Heck arylation of allylic alcohols in [Bu_4_N]Br.

A similar stabilization of the active Pd catalytic species was invoked by Muzart and co-workers, who employed NaHCO_3_ as the base and PdCl_2_ as the catalyst precursor in a Heck arylation of allylic alcohols in [Bu_4_N]Br, affording β-arylated carbonyl compounds (Scheme 6) [24].

**Scheme 7:**
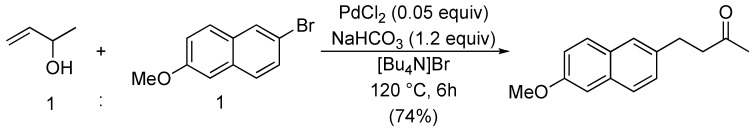
Synthesis of nabumethone.

This protocol, which let possible to recycle the active catalyst up to four times, was also applied to a one-step synthesis of the nonsteroidal antiinflammatory drug nabumethone (Scheme 7) [24].

**Scheme 8:**
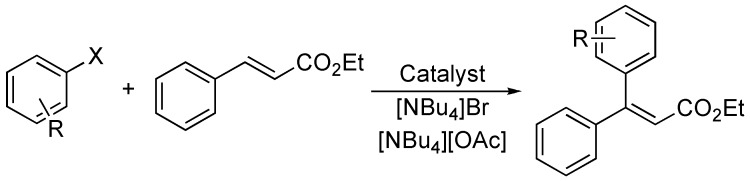
Heck arylation of ethyl *trans*-cynnamate.

During the past decade, in a series of important papers [25,26,27] Calo` *et al*. have evidenced the superiority of tetraalkylammonium halides over common pyridinium and imidazolium salts in terms of catalysts stability, reaction rates and regio- and stereoselectivity in Pd nanoparticles catalyzed coupling reactions. Bromoarenes were coupled with less reactive 1,2-disubstituted alkenes, such as *trans*-cinnamates, in a stereospecific manner under ligand-free conditions in 1:1.5 molar mixtures of tetrabutylammonium bromide and tetrabutylammonium acetate, TBAB-TBAA (Scheme 8) [26]. The observed stereoselectivity was ascribed not only to a better solubility of TBAA in TBAB, but also to an intramolecular neutralization of PdH, still ligated to the olefin, by an acetate ion in the metal coordination shell through a five-membered transition state. 

The absence of acetate in the coordination shell would allow the PdH isomerization, leading to a thermodynamic mixture of isomeric olefins. This palladium nanoparticles-catalyzed Heck arylation was extended to butyl methacrylate and α-methylstyrene. In this case, however, a 3:1 mixture of regioisomers was obtained in favour of the terminal olefins, together with variable amounts of double-arylated products [27].

**Scheme 9:**
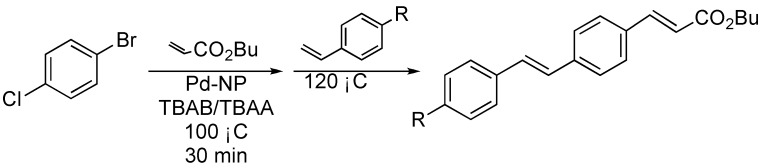
One-pot sequential coupling of 1-bromo-4-chlorobenzene.

In this contest, recently Nacci *et al*. have recently reported [28] a general method for coupling of aryl chlorides, including deactivated and electron-rich aryl chlorides, using ligand-free Pd(OAc)_2_ in the molten mixture of tetraalkylammonium ionic liquids (TBAB-TBAA) under aerobic and relatively mild conditions (Scheme 9). Interestingly, using this system it was possible to couple 1-bromo-4-chlorobenzene with two different olefins in a one-pot sequential manner by activating the C-Br and C-Cl bonds on the aromatic ring at two different temperatures of 100 and 120 °C. Unsymmetrical substituted arenes were produced with high reaction rates and high overall yield. 

**Scheme 10:**
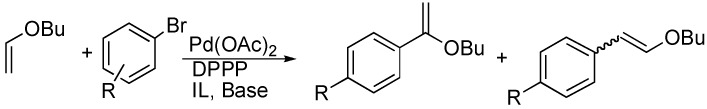
Highly regioselective arylation of electron rich olefins.

An extensive investigation on Pd-catalyzed Heck reactions in imidazolium based ILs has been performed by the research group of Xiao. In 2000, they published [29] a detailed study on reaction of iodobenzene with styrene and acrylates catalyzed by Pd(OAc)_2_, showing for the first time that the imidazolium cation can react with a catalyst precursor to form *N*-heterocyclic carbene complexes *via* deprotonation in the imidazolium-based ionic liquids under catalytic conditions, and that the carbene complexes so generated are active for C-C bond coupling reactions. Subsequently, they focused on the palladium catalyzed arylation of the electron-rich olefins, such as butyl vinyl ether in [bmim][BF_4_], using aryl iodides and bromides as the arylating agents. The results of these studies, reviewed in 2008 [30], showed that imidazolium ILs in combination with the readily available Pd(OAc)_2_ and 1,3-bis-(diphenylphosphino)propane (DPPP) form an excellent catalytic system, in which highly regioselective and yielding arylation of electron reach olefins can be performed with a wide range of aryl halides with no need for any halide scavengers (Scheme 10). 

Generally, the arylation gives only the branched olefins; the unique stereocontrol has been attributed to the ionic environment, which alters the reaction mechanism favoring the ionic pathway. It is however noteworthy that for this kind of reactions little conversions (<1%) were observed [31] in [bmim]Br and [bmim]Cl and these results were attributed to the coordination of halides with palladium and/or the formation of inactive imidazolium carbene complexes of Pd. The inhibitory effect of bromide on this reaction has been subsequently more extensively investigated [32] by the same authors, who on the basis of the dramatic decreases in the arylation rate with increasing bromide concentration, hypothesized the presence of an equilibrium (eq. 1) before the rate determining step.


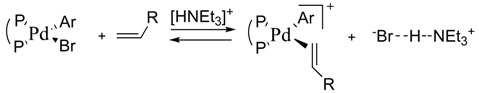
(1)

HBr, generated from each arylation, must be effectively scavengered by the NEt_3_, which may be able to trap also the bromide anions by hydrogen bonding between [HNEt_3_]^+^ and Br^-^. A remarkable accelerating effect exerted by the potential hydrogen bond donor [HNEt_3_]^+^ was evidenced in the same paper. 

In conclusion, whereas halides ([NBu_4_]Cl) are able to accelerate the Heck reaction employing palladium catalysts containing monodentate olefins or no ligands, they have an inhibitory effect on Pd-dppp catalyzed reactions. To a different mechanism, molecular in the first case and ionic in the second one, was attributed the different behavior (for a detailed discussion see below). 

**Scheme 11:**
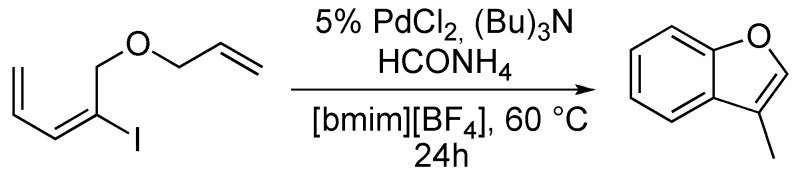
Intramolecular Heck reaction.

Despite of the extensive work performed on Heck reactions in the last ten years, it is however noteworthy that only few data have been reported on the intramolecular process. The sole paper on this topic evidenced that substituted benzofurans can be obtained [33] in modest to good yields by palladium-catalysed intramolecular Heck reaction in [bmim][BF_4_] (Scheme 11). In general, the yields of the substrates with a substituent on the aryl group were lower, although the character of the substituent was not able to affect significantly the reaction yield. The catalyst in the IL phase could be re-used by the addition of another portion of substrate, Bu_3_N and HCO_2_NH_4_; the catalytic activity varied from 71 to 57% after four cycles.

**Scheme 12:**
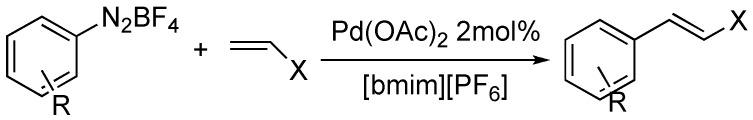
Heck reaction with arenediazonium salts.

Finally, it must be remarked that although generally aryl halides have been used in most of the investigated reaction in ILs, in 2004 Kabalka *et al*. reported [34] the palladium catalysed reaction of methyl acrylate and methyl acrylonitrile with arenediazonium salts in [bmim][PF_6_]. The reactions could be performed at room temperature for methyl acrylate and at 50 °C for acrylonitrile in the absence of base and in relatively short reaction times (Scheme 12). The catalytic system was recycled at least four times without loss of activity, although electron-rich olefins did not react and styrenes produced dimerisation products.

## 3. Task-Specific Ionic Liquids (TSILs) in the Heck Reaction

On the base of the data reported in the previous section it is possible to conclude that the Heck reaction based on the use of ILs is clearly advantageous from the point of view of product separation and re-use of the catalytic system. In addition, most of the reactions can be performed in the absence of added phosphorous containing ligands and, selecting ionic liquid and reaction conditions, it is possible to use also aryl bromides and chlorides or benzoic anhydrides. However, common ILs still have a tendency to leach dissolved catalysts into the co-solvent used to extract the product(s). To avoid metal catalysts leaching out of the IL system, significant efforts have been made to enhance the solubility of the catalysts in ILs and practically two approaches have been followed: i) functional groups able to coordinate with metal centers have been inserted into the ILs, ii) imidazolium/pyridinium tags have been introduced into a metal complex.

**Scheme 13:**
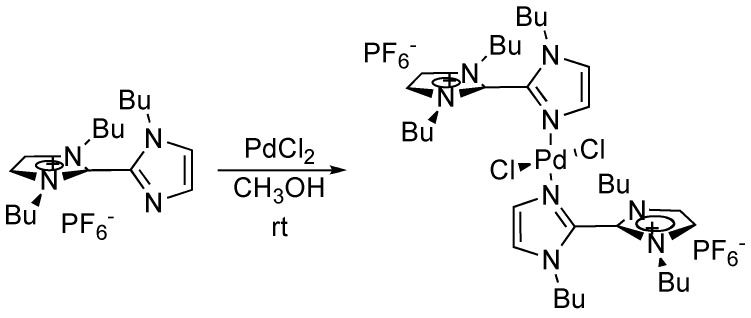
Palladium complex from 2-imidazole fuctionalized ILs.

In this context, in 2004 Shreeve *et al*. reported [35] the synthesis of the monoquaternary product arising from the reaction of 2,2’-diimidazole with iodobutane and the application of the obtained IL as solvent and ligand for the Heck reaction. The new palladium complex, reported in Scheme 13, was isolated by adding PdCl_2_ to this ionic liquid in methanol. The same 2-imidazole functionalized IL was used for the Heck coupling of iodobenzene with methyl acrylate in the presence of PdCl_2_ (2 mol%) and a Na_2_CO_3_ as a base. After product recovery (92% yield) the system was washed with water and re-used another four times without significant activity loss. The recovered system was used also with the non-reactive chlorobenzene, leading to the same product in 74% yield after two runs.

**Scheme 14:**
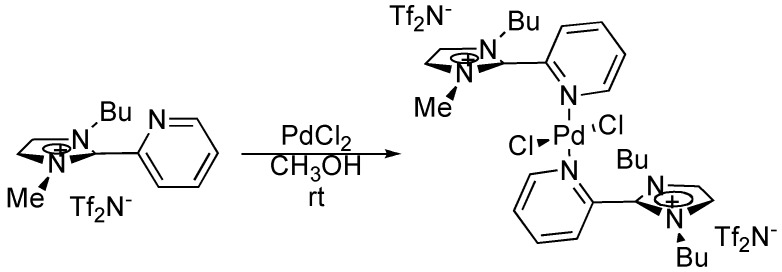
Palladium complex from 2-(2-pyridyl) fuctionalized ILs.

An analogous functionalized palladium(II) complex was obtained by reaction 1-butyl-2-(2-pyridyl)-3-methylimidazolium bistriflimide with PdCl_2_ in methanol. The coupling reaction of iodobenzene with butyl acrylate in the corresponding IL, 1-butyl-2-(2-pyridyl)-3-methylimidazolium bistriflimide, or in the analogous 1-butyl-2-(phenyl)-3-methylimidazolium bistriflimide could be performed successfully 10 times without detectable loss of catalytic activity (Scheme 14).

However, when the Heck reaction was carried out using PdCl_2_ under similar conditions, the formation of palladium black was evidenced and after two cycles the system became practically inactive. Moreover, whereas no appreciable difference in yields between activated and deactivated aryl iodides was found, the electronic nature of aryl bromides had a clear effect on the coupling reaction.

**Figure 1:**
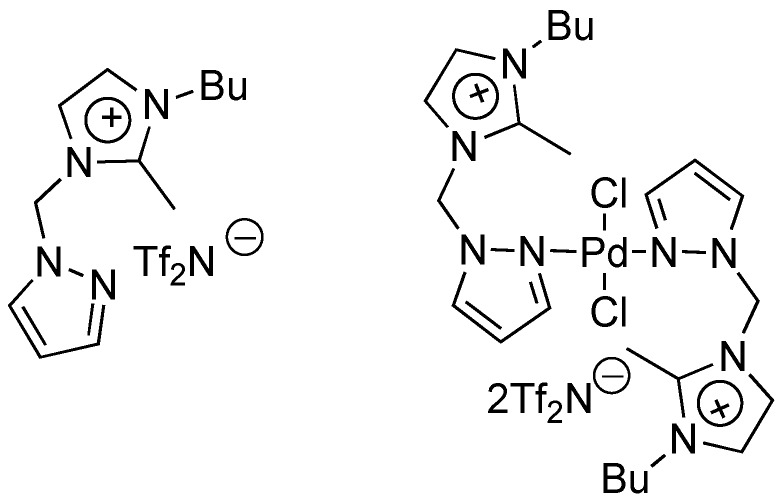
Palladium complex from N-pyrazolyl 2-methyl imidazolium based IL.

But the functionalization of the imidazolium ring not necessarily have to involve the C(2) imidazolium carbon. Substituents able to interact with palladium(II) have been introduced also on the imidazolium nitrogen. Pyrazolyl-functionalized 2-methylimidazolium based ILs have been therefore synthesized and the activity and recyclability of the palladium complexes have been evaluated [36] (Figure 1).

Initially, the Heck reaction was performed using as catalyst the functionalized palladium complex dissolved in the pyrazolyl-functionalized IL. However, interestingly, this catalyst could be used also in a unfunctionalized IL (1-butyl-2,3-dimethylimidazolium bistriflimide) obtaining at the 9th recycle a complete conversion of iodobenzene. The efficiency of the process was attributed to the presence on the catalyst of a pendant 2-methylimidazolium tag which is similar to the unfunctionalized IL and favour immobilization. Of course, when the Heck reaction was examined using PdCl_2_ as the catalyst precursor in the pyrazolyl-functionalyzed IL under identical conditions similar results were obtained. In this reaction, reactivity decreased using bromobenzenes whereas only traces of the product were obtained with 4-chlorobenzene.

**Figure 2:**
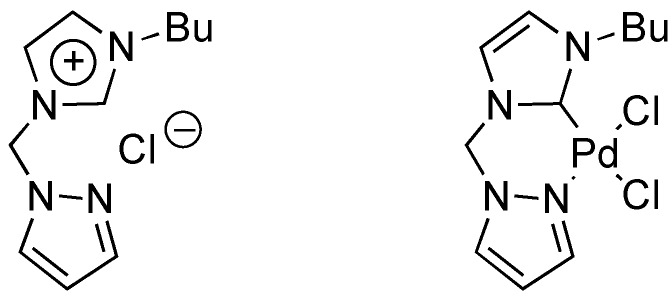
Palladium complex from N-pyrazolyl imidazolium based IL.

Starting from N-alkylimidazoles another series of pyrazolyl-functionalized imidazolium based ILs was subsequently synthesized and characterized [37]. Through a (carbene)silver complex, a palladium complex was prepared, isolated and characterized. In this case, as a consequence of the absence of the methyl group at C(2) on imidazolium ring, the functionalized IL acted as a chelating ligand coordinating the palladium(II) center through its carbene carbon atom and the pyrazolyl nitrogen atom to give a six-membered metallacycle with boat-like conformation (Figure 2).

**Figure 3:**
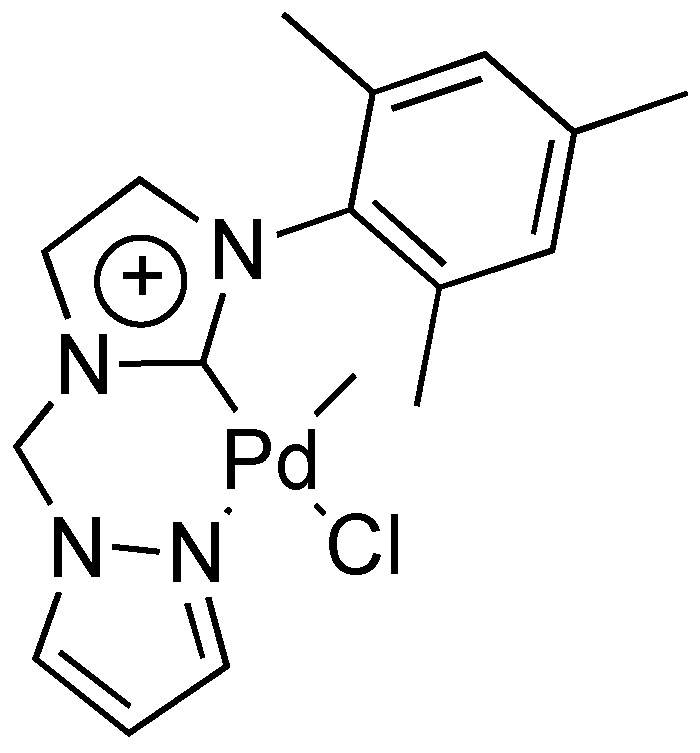
Palladium complex from N-pyrazolyl-N-aryl imidazolium based IL.

The catalytic activity as a pre-catalyst was investigated in the corresponding IL using the above reported procedure. Aryl halides gave with butyl acrylate the expected product in > 90 % yield independently of the substituents on the phenyl ring. No detectable loss of catalytic activity was observed after five recycles. Contemporaneously, also the activity of a hemilabile pyrazolyl-functionalized N-heterocyclic carbene complex of Pd(II) in [bmim][PF_6_] was evaluated by the same group [38]. The catalyst could be recovered and recycled at least three times without significant loss of activity (Figure 3).

**Scheme 15:**
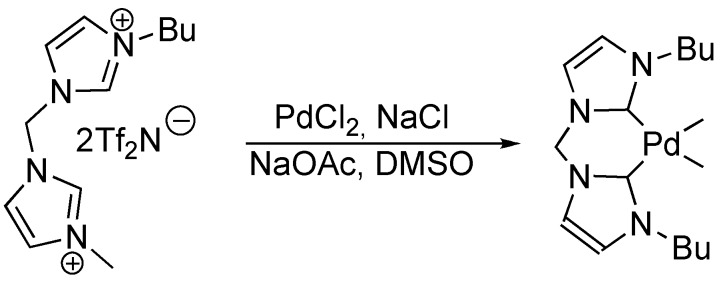
Palladium complex from an IL containing a pincer dication.

On the other hand, considering that some chelate or pincer N-heterocyclic Pd-carbene complexes exhibit high catalytic activity for Heck reactions [39], the application of an IL containing a pincer dication substituted with two alkyl chains has been investigated in the Heck reaction of aryl halides with butyl acrylate at 120 °C [40] by Shreeve *et al*. (Scheme 15). Iodobenzene was found to have the highest reactivity, giving *trans*-butylcinnamate in 92% yield using both PdCl_2_ or the isolated palladium complex as catalyst and NaOAc as base, whereas bromobenzene resulted in 71% yield and chlorobenzene was practically unreactive (3% yield). In this case, the recovered IL could be reused three-times without significant loss of activity.

Since the introduction of a third nitrogen atom on the five member heteroaromatic system could increase the acidity of the C-2 position favoring Pd-carbene complex formation, unsymmetrical dicationic salts incorporating imidazolium and triazolium functionalities were also synthesized and tested [41].

**Scheme 16:**
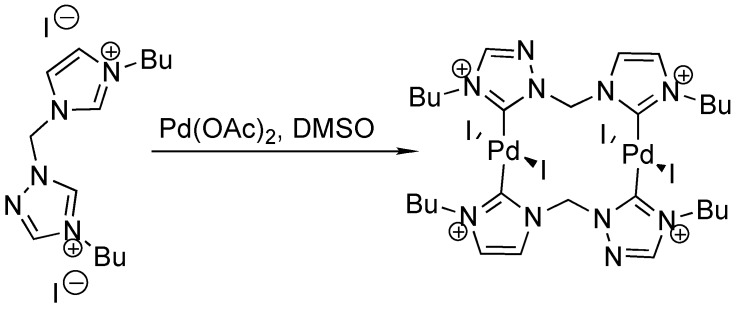
Synthesis of dinuclear palladium complex.

It is noteworthy that, instead of a chelating palladium(II) dicarbene complex, the reaction with Pd(OAc)_2_ in DMSO gave a dinuclear complex, which analogously to the above reported complexes could be used as pre-catalyst in the Heck reaction (Scheme 16). Comparable results were obtained using the same procedure.

Contemporaneously, the strategy to introduce specific functional groups on the alkyl chain of imidazolium cation to increase the catalyst immobilization has been followed by other research groups.

**Figure 4:**
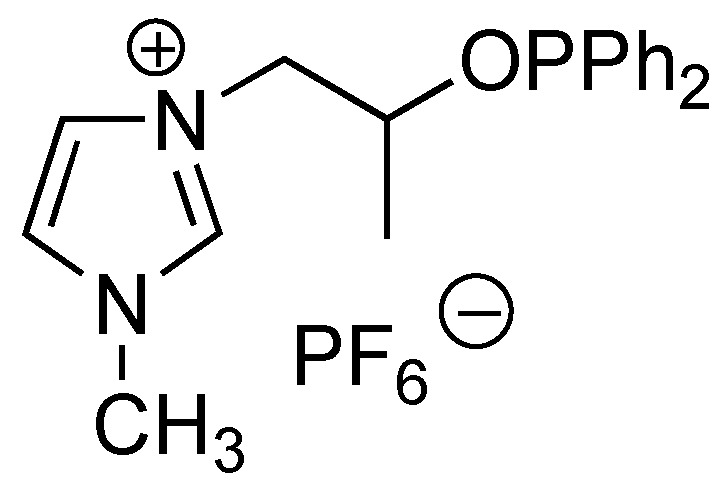
Imidazolium based phosphinite IL.

In this contest, it has been shown [42] that the use of an imidazolium based phosphinite IL (Figure 4) as solvent and as ligand in the coupling reaction of aryl halides (including chlorobenzene) with styrene and butyl acrylate allows the synthesis of the expected product in high yield (generally, around 90% also for ArCl) assuring also a high recyclability (six runs without losing its efficiency). Although the crystal structure of the catalyst was not reported, the formation of a carbene-type complex was excluded on the basis of the ^1^H NMR spectrum, and a ML2 complex was hypothesized.

**Figure 5:**
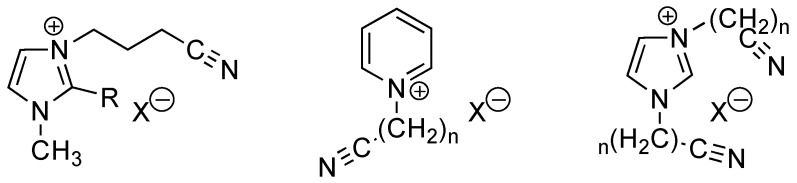
Nitrile functionalized ILs.

Significant results have been obtained also by Dyson *et al*. introducing a nitrile group on the alkyl chain(s) of pyridinium and imidazolium based-ILs (Figure 5) [43,44,45]. 

**Figure 6:**
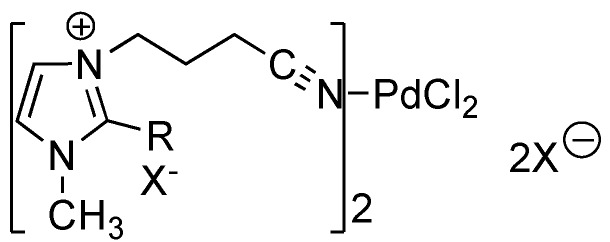
Coordination of the nitrile group to the palladium center

Nitrile-functionalized ILs have been used with success in several palladium catalyzed reactions, including the Heck process and their usefulness became apparent upon catalyst recycling; while in conventional ILs activity rapidly decreased to zero, in nitrile-functionalized ILs little change was observed after several recycles. Functionalization of the alkyl chain facilitates the solubility of the Pd(II) precatalyst *via* weak coordination of the nitrile groups to the palladium center, as evidenced through IR measurements (Figure 6). Moreover, when palladium nanoparticles are involved nitrile group may form a protective sheath around the nanoparticles, preventing aggregation.

It is noteworthy that, at variance with other palladium catalyzed reactions, reuse of the IL system in the Heck reaction was not very encouraging, due to observed progressive loss of activity. The decrease in reactivity of the catalyst could be however ascribed to the consumption of the base; the addition of 1 equiv of cholinium acetate after the fourth cycle restored the reactivity of the system.

**Figure 7:**
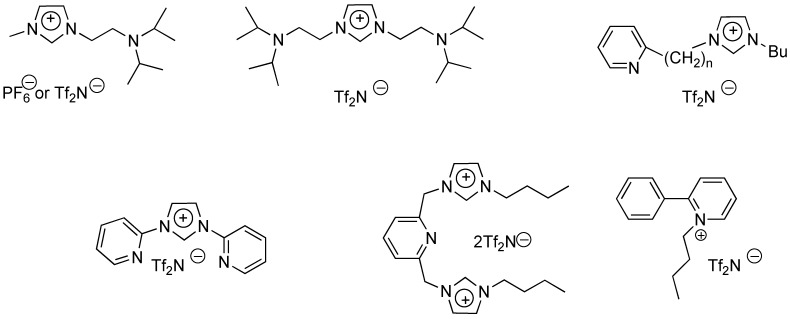
Basic ionic liquid cations.

To overcome the problems arising from the depletion of the base during the recycles, more recently basic IL have been employed as both solvents and proton scavenger in Heck coupling reactions. The first example, reported [46] by Shreeve *et al.*, evidenced that IL bearing tertiary aliphatic amines are effective media for the Heck reactions (Figure 7). 

Under reflux conditions, iodobenzene reacted with butyl acrylate to give the cinnamic ester in 100% yield. The catalytic system could be reused simply by washing with NaHCO_3_; after five recycles no activity loss was observed. Also in this case the formation of palladium nanoparticles was observed; all attempts to synthesize carbene-palladium complexes resulted in stable palladium colloids. The hypothesis that the tertiary amine might act as reducing agents in the redox process, leading to the formation of nanoparticles, was advanced.

**Figure 8:**
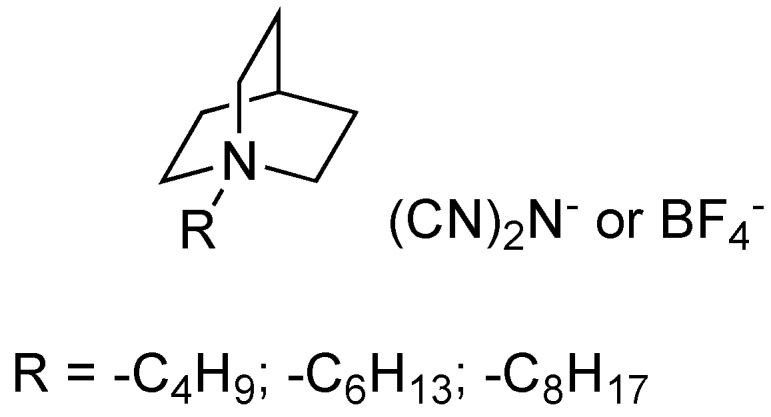
1,4-Diazabicyclo[2,2,2]octane (dabco)-based ILs.

Subsequently, 1,4-diazabicyclo[2,2,2]octane (dabco)-based ILs (Figure 8) having as counteranion the basic dicyanamide or tetrafluoroborate have also been tested in the Heck reaction [47]. Although after two hours the yields of the recovered product for the model reaction (iodobenzene-ethyl acrylate) were lower than in the comparative reaction performed in [bmim][BF_4_], the dabco-based ILs could be reused without re-addition of base and reducing agent.

**Figure 9:**
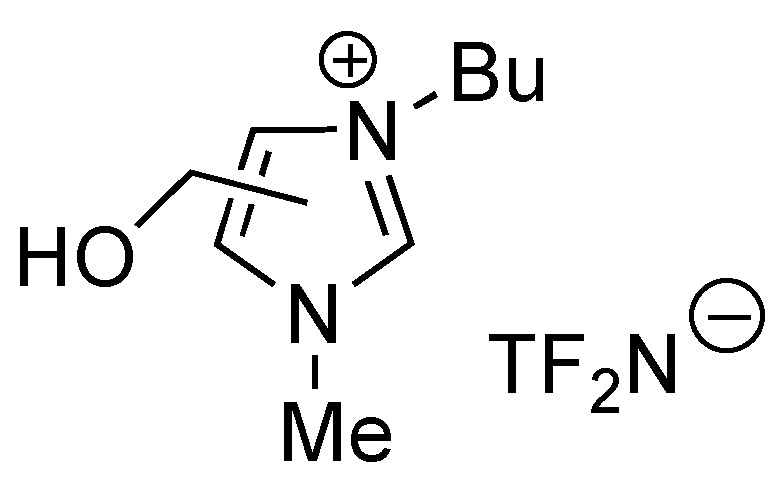
Hydroxyl functionalized ILs.

Finally, also hydroxyl or diol functionalized ILs (Figure 9) have been employed has reaction media and catalyst ligands in the Heck reaction. In 2003, Handy *et al*. prepared a new class of hydroxymethyl substituted imidazolium based ILs using fructose as the starting material [48]. 

The ionic liquid having bistriflimide as counteranion was employed as solvent for the model Heck reactions of methyl acrylate with simple aryl iodides in the presence of Pd(OAc)_2_. The coupling products were isolated in almost quantitative yields simply by extracting the crude mixtures with cyclohexane. Moreover, the solvent and catalyst were recycled 4-5 times without affecting the efficiency of the reaction. Also in this medium, however, the Heck coupling was slower than those performed in the standard, non-protic [bmim] series ionic liquids, although not at a great extent. An interesting accelerating effect of catalytic amounts of certain halide ions on the coupling of iodobenzene and methyl acrylate was observed.

**Figure 10:**
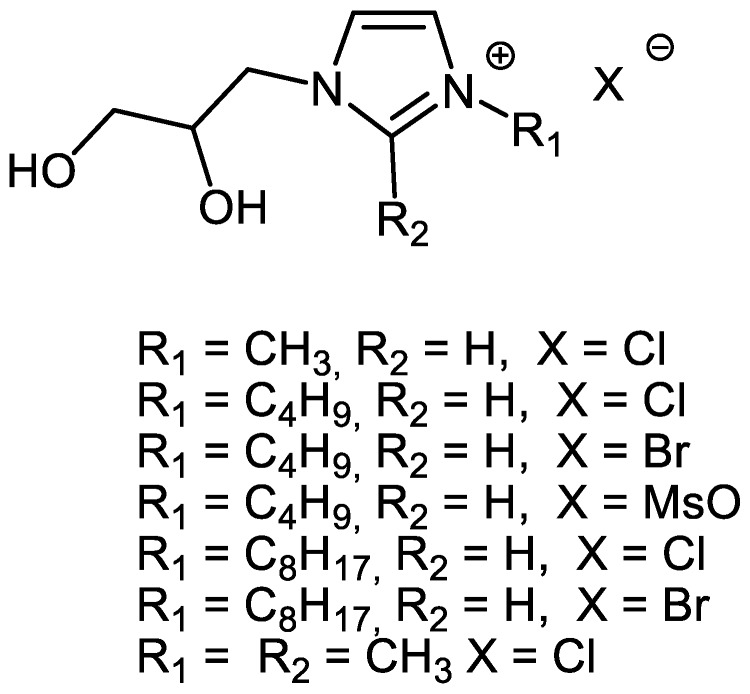
2,3-Dihydroxyl functionalized ILs.

More recently, a series of imidazolium based ILs N-functionalized with a 2,3-dihydroxypropyl group (Figure 10) has been used as ligands and solvents for palladium(II)-catalyzed reactions; these compounds present, besides the N-heterocyclic donor moiety arising from the imidazolium skeleton, the ethylene glycol group able to act as an excellent chelating ligand [49]. 

The Heck coupling of iodobenzene and ethyl acrylate in glycerylimidazolium-based ionic liquids at 100 °C has been performed in the presence of 5 mol-% PdCl_2_ as the catalyst precursor and AcONa (1.1 equiv) as the base. Although reuse of the IL systems was not very encouraging, due to the progressive loss of activity, a high reaction rate was observed in particular in 1-glycerol-3-octylimidazolium chloride, [GLYOCTIM]Cl. The reaction yields increased on increasing the alkyl chain on cation ([GLYOCTIM]Cl > [GLYBIM]Cl > [GLYMIM]Cl, and [GLYOCTIM]Br > [GLYBIM]Br) and were affected by anion nature and by the presence of a methyl group at C(2). The very low yield obtained by using 1,2-dimethyl-3-glycerylimidazolium chloride as solvent suggested that imidazolium carbenes, formed by reaction of the base with the acidic C(2)-H bond of the [GLYIM^+^] cations, might be actively implicated in these reactions (*vide infra*). Since the formation of carbenes in imidazolium-based ILs should be favoured by the basicity of the counteranion [50], the involvement of these species is also in agreement with the anion effect: bromides give lower conversions than chlorides and practically no reaction was observed in [GLYBIM]OMs. 

Finally, 1-glycerol-3-methylimidazolium hexafluorophosphate has been successfully used [51,52] also as the active and recyclable catalytic system in the reaction of aryl bromides with methyl ethyl acrylate under aerobic condition in DMF. 

**Figure 11:**
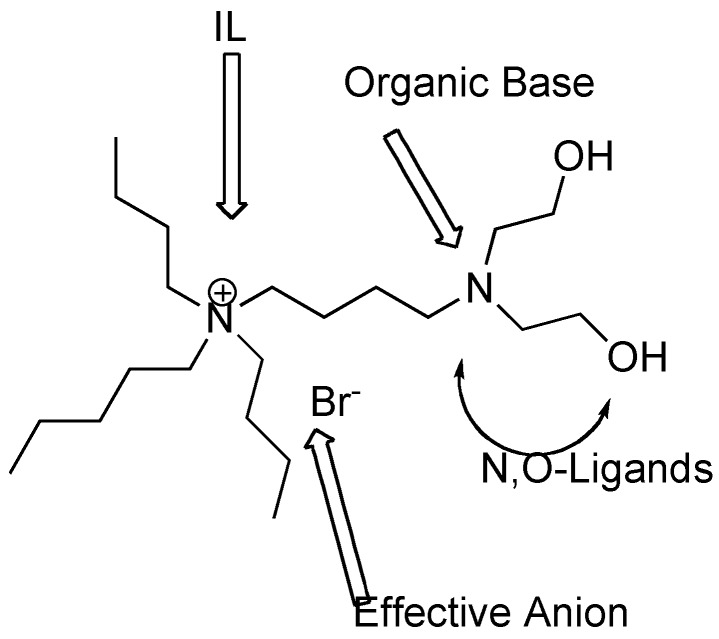
Multifunctionalized IL.

Recently, a novel IL able to act as base, ligand and activator (Figure 11) has been synthesized by Wang *et al*. [53] The olefination process of alkenes (styrene, acrylates and acrylonitrile) with activated and deactivated iodo, bromo and chloroarenes has generated the corresponding products in good to excellent yields in 4-(dihydroxyethyl)aminobutylammonium bromide). Transmission electron microscopy (TEM) has evidenced the formation of particles having an average diameter of *ca.* 4 nm suggesting that the ethanolamine functionalized IL are able to stabilize palladium colloids. It is noteworthy that the system is characterized by a high recyclability; palladium and IL were recycled six times with no loss of their activity and the inductively coupled plasma (ICP) analyses of the extracted organic solvents indicated that Pd content was < 0.2 ppm. 

**Figure 12:**
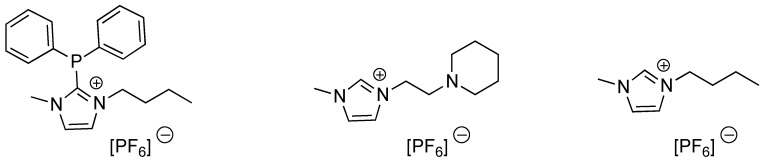
Mixture of monofunctionalized ILs.

An alternative approach to the use of multifunctionalized ILs may be the use of mixtures of differently substituted ILs which can exert the functions of ligand, base and reaction medium as one unit. In this contest, the ternary mixture of 1-butyl-2-diphenylphosphino-3-methylimidazolium hexafluorophosphate, [bdppmim][PF_6_], 1-(2-piperid-1-yl-ethyl)-3-methylimidazolium hexafluorophosphate, [pemim]PF_6_] and 1-butyl-3-methylimidazolium hexafluorophosphate, [bmim][PF_6_] represents an efficient and recyclable system for the PdCl_2_ catalyzed coupling of aryl halides (including hindered and electron-rich aryl iodide and activated aryl bromides) with ethyl acrylate (Figure 12) [54].

Experiments performed using the single ILs or their binary mixtures evidence a synergistic effect when used as one unit: due to the presence of more coordinating sites the palladium catalyst combined the advantageous activity and stability of N,P-containing ligand coordinated Pd complexes and Pd-carbene complexes, although the exact nature of the species present in solution was unknown. Furthermore, the strong hydrophobicity of the resulting system allowed the recovery of [pemim][PF_6_] during the washing phase between two subsequent cycles and the recycling uses of palladium catalyst.

More recently, the catalytic activity of 1-butyl-2-diphenylphosphino-3-methylimidazolium hexafluorophosphate, [bdppmim][PF_6_], or 1-(2-piperid-1-yl-ethyl)-3-methylimidazolium hexafluorophosphate, [pemim][PF_6_] in the model Heck reaction of aryl halides with methyl or ethyl acrylates in [bmim][PF_6_] has been compared [55] with that of a hybrid P,N ligand functionalized IL, 1-(2-piperid-1-yl-ethyl)-2-diphenylphosphino-3-methylimidazolium hexafluorophosphate, [pedppmim][PF_6_], combining the structural peculiarities of the other two ligands in a sole molecule. In this case an organic base was always added. 

With the involvement of the hybrid P,N-ligand, PdCl_2_ exhibited a very good activity and stability also after seven runs without any precipitation of black palladium. In each run pure ethyl cinnamate was obtained in >96% yield, whereas the ICP analysis indicated that leaching of Pd in the organic phase was below the detection limit (< 0.1 mg/g). When the sole P-containing ligand was used lower yields were obtained although good stability was observed. At variance, in the N-containing ligand the palladium catalyst deactivated gradually during the recycle procedure with the obvious precipitation of Pd black, though its fresh activity was better than the corresponding hybrid P,N-ligand. Therefore, as suggested by the authors, the results seem to show that as a bidentate ligand, the P,N-functionalized IL, with a hemilabile ligation to metal center could offer the necessary insaturation site for the substrate (at N-coordinating site) simultaneously holding the Pd by the P-ligand arm. Moreover, the bonding of the phosphine moiety to C-2 position of the imidazolium, having an electron-withdrawing nature, results in a decreased electron density at the phosphorous center, leading to an improved oxidation tolerance of its self with consequent stability of the catalyst. This feature was evidenced also for the P-containing ligand in the above discussed ternary mixture.

**Figure 14:**
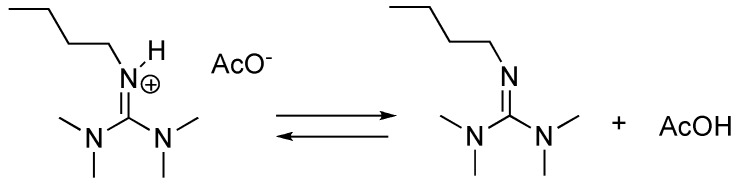
Brønsted guanidine acid-base ionic liquid.

Finally, although many of the works on Heck reaction are related to imidazolium based ILs, in 2006 an interesting paper highlighting the possibility to the use of a Brønsted guanidine acid-base ionic liquid as media and catalyst was published (Figure 14) [56].

The basic butyltetramethylguanidinium acetate, prepared by neutralization of the corresponding guanidine with acetic acid, was used successful as solvent, ligand and base in the reactions of aryl halides with styrene and butyl acrylate at 140 °C. High yields of the expected products were obtained in subsequent runs (the system was reused five times) until all acetate was converted to bromide. Determination of the palladium that remained in IL evidenced only a slight decrease. It is noteworthy that quantitative conversion was achieved within 0.25 h when 0.16 mol % of PdCl_2_ was used; decreasing the catalyst loading to 0.01% still resulted in quantitative conversion although the reaction time was prolonged at 20 h.

**Figure 15:**
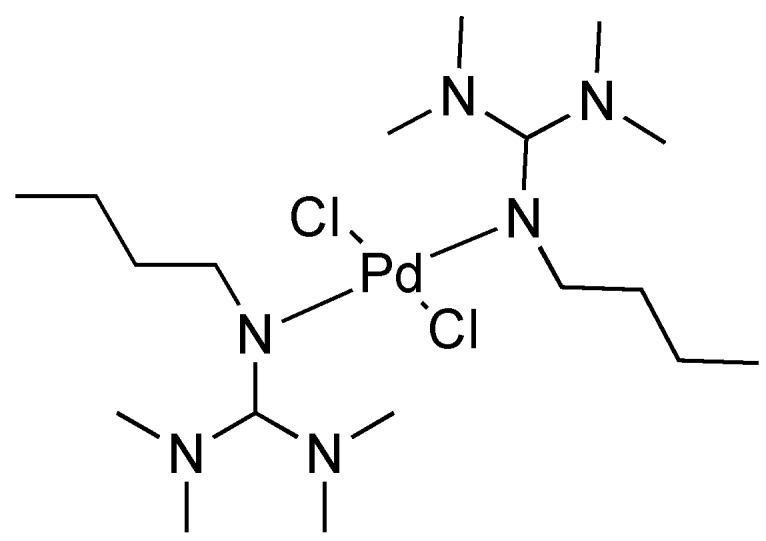
The molecular structure of the Guanidine_2_PdCl_2_ complex.

Considering that at elevated temperature the guanidinium based IL may dissociate into guanidine and acetic acid, the author suggested that the generated guanidine acts as a base and a ligand for active Pd species. The molecular structure of the corresponding L_2_PdCl_2_ complex was reported (Figure 15) and its activity as catalyst for the reaction between bromobenzene and styrene was tested in DMA.

In conclusion, this simple IL is able to play multiple roles in this process: i) it acts as ligand stabilizing the activated Pd(0) during the reaction; ii) it offers a strong base to favor the β-hydride elimination; iii) it acts as a polar solvent increasing the reaction rate. Unfortunately, no other data or example of application of this kind of IL has been subsequently reported.

## 4. Heterogeneous Heck Reaction in ILs 

To simplify processes and ensure reproducibility, process chemists generally gravitate toward reaction chemistry that can be run in a single common liquid phase. However, significant productivity advantages can frequently be achieved by employing multiple phases (*i.e.*, liquid-liquid, gas-liquid, solid-liquid). As reported in the previous sections, liquid-liquid biphasic Heck processes involving ionic liquids and organic product phases have been the subject of intensive investigation since the ionic catalyst phase often induces excellent catalyst immobilization and also low miscibility with the organic products. However, application of these procedures in industrial processes is still hampered by the fact that these methodologies use large amounts of ILs. Moreover, in many cases liquid-liquid biphasic catalysis uses only fraction of the IL and of the dissolved catalyst. Finally, catalyst leaching cannot be completely avoided.

**Figure 16:**
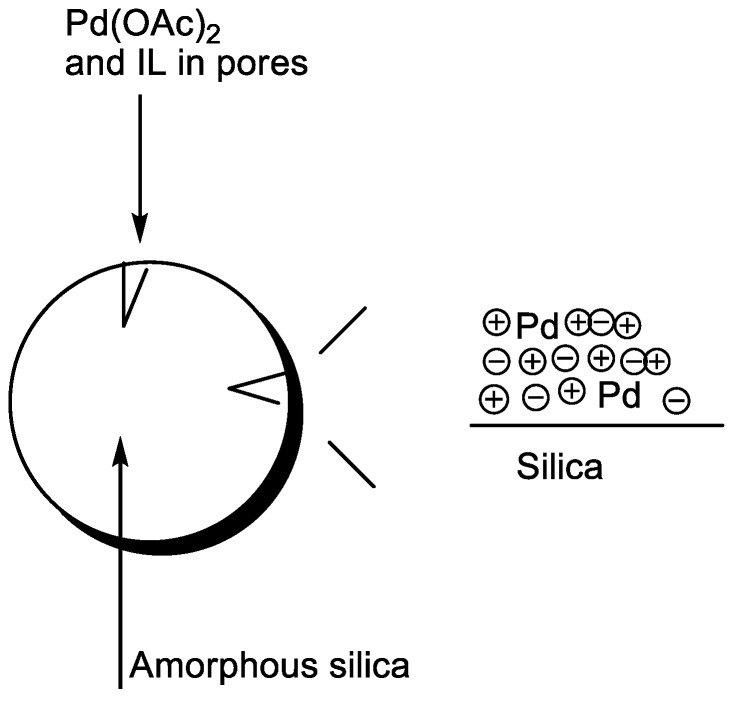
Immobilized Pd(OAc)_2_ in silica gel pores using [bmim][PF_6_].

To increase catalyst performance and develop more sustainable and environmental benign reaction conditions, in the last decade several attempts have been done to perform the Heck reaction under heterogeneous conditions. The concept of confining a catalyst on porous supports is far from new, and several palladium catalyst reservoirs are commercially available; these systems have tested also in ILs. In 2001, the use of Pd/C as a stable and inexpensive catalyst in [bmim][PF_6_] in the presence of Et_3_N was reported [57]; the procedure assured the re-cycle of the catalytic system without loss of activity. Moreover, ICP analysis of the ionic liquid performed before and after reaction revealed that the concentration of Pd in the ionic medium was negligible, suggesting that the Pd/C catalyzed reaction occurs on the surface of Pd held on the carbon. More recently, the same research group has reported [58] the very facile and economically friendly immobilization of Pd(OAc)_2_ in silica gel pores, with the aid of [bmim][PF_6_], and the application of this catalytic system in recyclable Heck reactions (figure 16). The catalyst immobilization on the silica gel was highly effective in dodecane at 150 °C giving a 95% average yield and a TON of 68,000 up to 6th use. The use of a hydrocarbon solvent was however necessary to prevent removal of the IL layer from the silica. 

Promising results in terms of both yield and sustainability, were obtained through the immobilization of Pd(OAc)_2_ on reversed phase amorphous silica gels, always with the aid of [bmim]PF_6_. In this case, a water compatible catalytic system was obtained [59]. The Heck reaction of iodobenzene with cyclohexyl acrylate using this catalyst, carried out in water at 100 °C, was characterized up to the sixth re-use by an average 95% yield with TON and TOF 1,600,000 and 71,000 (h^-1^), respectively. Moreover, different by the reaction in dodecane, there is no need, for recycle use, to wash the recovered catalyst with alkaline solution to remove ammonium halide. It is noteworthy that when silica supported palladium complex catalysts were used for the reaction of iodobenzene with olefin also in [bmim][PF_6_], although the system exhibited higher activity that in DMF, a dissolution of the catalyst from the silica support to the medium during the reaction was evidenced [60]. This latter process, therefore, although mediated by supported metal Pd may be considered to proceed as a quasi-homogeneous process. 

Support, solvent and form of palladium catalyst determine the intrinsic nature of the process and the possibility to have or not the direct involvement of Pd atoms located onto the support into the catalytic cycle. In this contest, a recent study of the Heck reaction between bromobenzene and styrene performed in tributylhexylammonium bistriflimide, using ultrafine particles of palladium metal supported onto hydrophilized mesoporous soot as the catalyst, has confirmed the fundamental role of the IL in the catalyst stabilization, also evidencing that the reaction occurs through a “true heterogeneous” mechanism [61]. 

**Figure 17:**
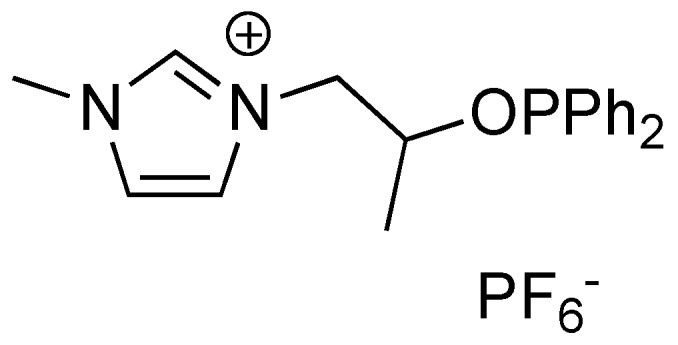
Phosphinite functionalized IL.

Highly efficient and stable supported palladium nanoparticles have been obtained also by contacting a solution of Pd(OAc)_2_ with an ionic liquid- modified xerogel [62], containing a phosphinite functionalized IL (Figure 17) attached to the silica matrix able to act as both as complexing and reducing agent.

It is noteworthy that Pd(OAc)_2_ solution gives by interaction with this matrix highly dispersed uniformly sized Pd nanocatalysts, tightly supported on the surface of the silica and not embedded in the bulk of xerogel.

However, sol-gel processing has been also used to encapsulate Pd(OAc)_2_ catalyst into a ionic phase-confined silica gels [63]. Pd-doped ionogel pellets have been employed in the coupling reaction of ethyl acrylate with iodobenzene using toluene as solvent. No significant difference in the reaction rate, when compared to homogeneous system, was observed, whereas leaching tests showed that catalysts actually took place in the IL phase confined within the silica matrix.

But, silica and carbon are not the sole supports used. Palladium nanocolloids supported on chitosan have been found to be an efficient and recyclable catalyst for Heck arylation of alkenes using tetrabutylammonium bromide (TBAB) as solvent and tetrabutylammonium acetate (TBAA) as base [64]. The efficiency of this catalyst has been attributed to the stabilization of palladium colloids by the solvent and to a very fast PdH neutralization by the base. It is noteworthy that the conversion rate decreases significantly in imidazolium based IL (including [bmim]Br) whereas in TBAB recyclability is possible only using aryl iodides; when bromobenzene was used the efficiency of the catalyst decreased with a concomitant leaching of the palladium from the complex, attributed to a decomposition of TBAB at 130 °C (temperature necessary for reaction of bromobenzene) which destabilizes the nanocomposite.

Recently, also Pd nanoparticles immobilized on molecular sieves SBA-15 (thiol-modified mesoporous materials) using 1,1,3,3-tetramethylguanidinium lactate have been synthesized [65]. The Heck arylation of olefins using this catalytic system was carried out under solvent-free conditions; the model reaction, iodobenze- methyl acrylate proceeded smoothly and the yield of *trans*-methyl cinnamate was 93%, even when the amount of Pd was 0.001 mol%. Moreover, no deactivation of the catalyst was observed after six repeated catalytic coupling reactions. It is noteworthy, that the reusability of the Pd catalyst supported on SBA-15 in the absence of the IL was significantly lower; it became an inactive white power after two recycles. The different behavior, with and without IL, has been attributed on the basis of transmission electronic microscope (TEM) images to the different size and distribution of the catalyst. In the presence of the IL, most of the Pd nanoparticles had before use a diameter in the range of 3-6 nm and they existed in the channels of the support. At variance, in the absence of the IL, the diameters of most Pd nanoparticles were in the range of 9-12 nm, bigger that the pore diameter of SBA-15, resulting in their existence on the outside surface of SBA-15. After, six recycles, most Pd nanoparticles on IL containing SBA-15 still exist in the channels, although some bigger Pd particles were formed, whereas in the absence of IL already after 2 recycles, most of the Pd disappeared and few Pd particles existed on the surface of the support. But, interesting results related to the catalytic mechanism of these systems have been obtained through filtration tests. The solid-free filtrate, obtained after separation of the catalyst before the end of the reaction, was able to continue to react, suggesting that the leaching of the active palladium species from the solid support occurred during the coupling. However, Pd re-deposited back onto the support after the end of the reaction. 

**Figure 18:**
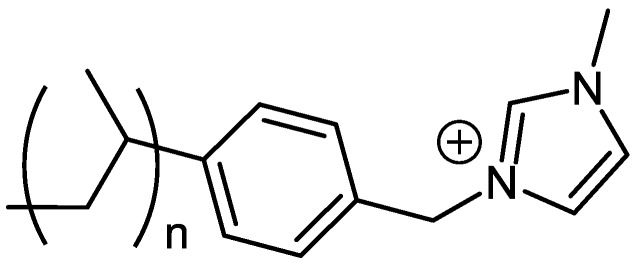
Imidazolium-based polymers.

Among the possible supports for Pd nanoparticles ionic polymers also have to be mentioned. A number of imidazolium-based polymers have been indeed synthesized (Figure 18), including poly(3-(4-vinylbenzyl)-1-methylimidazolium chloride and bistriflimide [66], and their ability to stabilize metal nanoparticles has been tested in coupling processes, including the Heck reaction [67].

In particular, it has been shown that highly stable Pd nanoparticles, protected by an imidazolium based ionic polymer in a functionalized IL, can be easily prepared by reduction with NaBH_4_. These viscous systems, containing Pd nanoparticles, are excellent pre-catalysts for Suzuki, Heck and Stille coupling reactions and can be stored without undergoing degradation for at least two years; compared to commercial Pd/C systems that typically required Pd loading of 2–4 mol % loading of 0.5–1.0 mol % can be employed using these systems. 

**Scheme 17:**
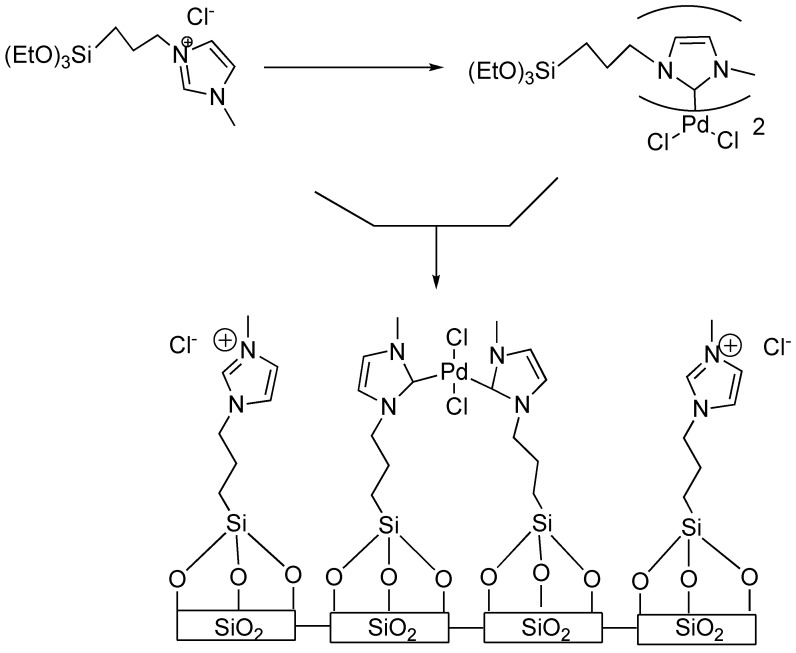
N-heterocyclic carbene palladium/IL matrix tethered on the silica surface.

In all the above discussed systems the Pd catalyst is held in/on a more or less porous solid by physic absorption, sometimes using the ability of the IL to coordinate the active catalytic species. However, immobilization can also be obtained through the covalent anchoring of the catalysts to the support surface [68]. Trimethoxysilylpropyl functionalized ILs may been anchored to silica, *via* the reaction of the appended alkyl–silyl side branch tethered into the anion or cation. Using this approach to graft imidazolium cations onto pre-dried silica, the subsequent treatment with PdCl_2_, gave non-acidic supported palladium-based ILs to use in Suzuki-Miyaura coupling reactions [69]. But the same approach was used also to tether a N-heterocyclic carbene palladium/ionic liquid matrix on the silica surface (Scheme 17).

The latter system, constituted by grafted N-heterocyclic carbene palladium complexes in grafted ILs, was effectively applied to the Heck reaction of a wide variety of iodo and bromoarenes. The catalyst showed high thermal stability (up to 280 °C) and could be recovered and reused for four cycles, giving a total TON = 36,600. It is noteworthy that, TEM coupled with EDX analysis indicated the formation of Pd nanoparticles with irregular shape and wide-range size distribution confined inside the irregular pores of amorphous SiO_2_.

**Figure 19:**
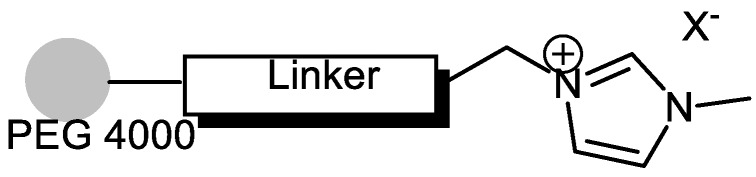
PEG supported ILs.

Finally, it is worth mention that polymeric beads of supported ILs (Figure 19), prepared *via* the covalent anchoring of an imidazolium salt to a PEG support, have also been used as a recyclable (100% yield after five cycles in the reaction of bromobenzene with styrene) catalytic system for the Heck reaction of aryl bromides and activated aryl chlorides [70]. Also in this case the drop in conversion starting from the sixth cycle was attributed to the accumulation of inorganic salts.

## 5. The Role of ILs in the Heck Reaction: the “Ionic Liquid Effect”

Ionic liquids constitute not only good solvents for the Heck reaction, due to their properties, but their unique ionic environment may change the course of the reaction, activating and/or stabilizing intermediates or transition states in the reaction mechanisms. As a consequence, performing the Heck reaction in these solvents may lead to an increasing in the reaction rate in comparison with “classical” solvents, to a stabilization of the catalytically active species and, in favorable cases, also to a control on the regio- and stereoselectivity of the coupling products. The nature of the ionic liquid however affects not only the kinetic constants of the single steps of this reaction but, probably, determines also the nature of the catalysts or pre-catalysts. The involvement of carbene complex, palladium nanoparticles and palladium anionic complexes seems to depend on the ionic liquid anion-cation structure.

### 5.1. ILs in ligand-free Heck reactions and their role in the stabilization of metal nanoparticles

**Figure 20:**
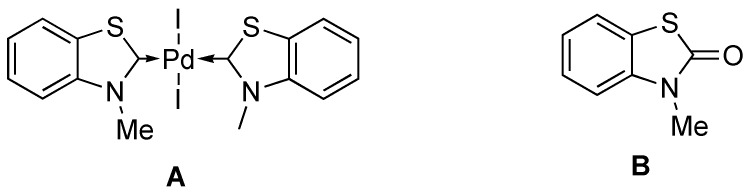
Carbene palladium precatalyst (A) and 2-oxo-3-methylbenzothiazole (B).

As reported above, the formation of palladium nanoparticles in the Heck reaction performed in ILs has been more times evidenced. Calò and co-workers described the use of Pd-NPs generated *in situ* by reaction of the carbene Pd precatalyst **A** in [Bu_4_N]Br using [Bu_4_N][OAc] as the base (Figure 20) [26,28]. They found that Pd-NPs are rapidly formed when [Bu_4_N][OAc] is added to **A** dissolved in [Bu_4_N]Br at 130 °C. In addition to the formation of a black suspension, which is typical when NPs are involved, the authors isolated 2-oxo-3-methylbenzothiazole (**B**) arising from deligation of the carbene ligands in **A** (Figure 20). 

Pd-NPs where confirmed by TEM analysis of a reaction sample [26]. Moreover, as already observed by Reetz and Westermann [71], Pd-NPs were also formed simply dissolving Pd(OAc)_2_ in [Bu_4_N]Br and adding [Bu_4_N]OAc, obtaining rapidly a dark dispersion [26].

Pd-NPs dispersed in [BMIM][PF_6_] have been prepared also by Dupont and co-workers and tested in the coupling of aryl iodides with *n*-butylacrylate using NEt(*i*-Pr)_2_ as the base in a temperature range of 30–130 °C [72]. The Pd-NPs before and after the coupling were analyzed by TEM, and the palladium content of the organic phase during the arylation reaction was checked by ICP-AS. The results obtained in this study were interpreted as strong indications that Pd-NPs dispersed in the ionic liquid act as a reservoir of catalytically active Pd species, and that very probably the reaction proceeds through the oxidative addition of PhI on the NPs surface, and the oxidized Pd species thus formed are detached from the surface and enter in the main catalytic cycle [72]. However, more recently this definition has been considered not formally correct. In fact, when the Pd(0) agglomeration rate is quite high there is an increase in the contribution of a second catalytic cycle to substrate conversion, and this make impossible a definition of metallic palladium as a catalyst reservoir [19].

**Scheme 18:**
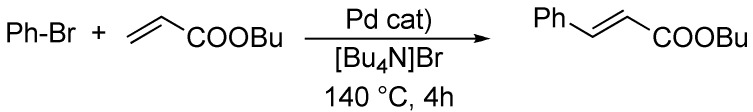
Base-free Pd-catalyzed Heck reaction in [Bu_4_N]Br.

The non-linear dependence of reaction yield on catalyst concentration has also been associated with the formation of Pd-NPs as the reactive form of the catalyst. In fact, initially the Pd(0), formed by a rapid reduction in situ of the Pd(II) precatalyst, is in the form of Pd clusters that are stabilized to some extent by the ionic liquid (for example, it has been suggested that in ammonium halides an “electrosteric” stabilization of metal NPs is operative: the halide anions provide electrostatic stabilization, and the ammonium cations a steric stabilization [73]). If the oxidative addition reaction is slow, the clusters may undergo ripening to form large crystals that precipitate as Pd black. Hence, to prevent this it may be better to increase the substrate/catalyst ratio [74]. As an example, in a recently published protocol for the base-free Pd-catalyzed Heck reaction in [Bu_4_N]Br, the yield of butylcinnamate formed by reaction of bromobenzene and butylacrylate strongly depends on the concentration of palladium. In particular, the maximum yield was obtained for PdCl_2_(PhCN)_2_ at 0.09 mol%, and for PdCl_2_ and Pd(OAc)_2_ at *ca*. 0.4 mol%, while lower yields were obtained below and over that concentrations (Scheme 18) [75].

As stated, a similar behavior has been interpretated as the effect of Pd-NPs formation, but the authors suggested that also a concomitant formation of less active dimeric or polymeric Pd(II) complexes could also be considered another explanation of the observed concentration effect [75].

**Figure 21:**
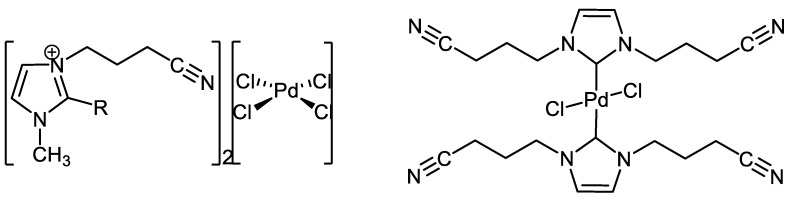
Palladium complexes with nitrile functionalized ILs.

Palladium nanoparticles have been evidenced also in functionalized ILs [43]. TEM images evidence that nanoparticles isolated from [bmim][BF_4_] and [C_3_CNmim][BF_4_] have a similar diameter of *ca*. 5 nm, but they show different morphologies. The nanoparticles obtained from nitrile functionalized ILs were well-separated, whereas those from [bmim][BF_4_] formed nanoclusters up to ca. 30 nm, suggesting that the nitrile group exerts a stabilizing effect and prevents aggregation. Nevertheless, in nitrile functionalized ILs the involvement of carbene complexes of the type, [(C_3_CN)_2_im]_2_[PdCl_2_] as catalysts or precatalysts has been excluded: Heck reactions performed using the pre-formed [(C_3_CN)_2_im]_2_[PdCl_2_] carbene complex were characterized by low conversions (ca. 2–8%), when carried out under conditions giving > 95% yields with PdCl_2_. It is noteworthy that not only PdCl_2_ but also [(C_3_Cdmim][PdCl_4_] can act as an active pre-catalyst in the Heck reaction. [(C_3_Cdmim][PdCl_4_] is formed by addition of PdCl_2_ to [C_3_CNmim]Cl; also in nitrile functionalized ILs if the anion is a strong nucleophile, such as chloride, the preferential coordination of the chloride anion with the metal takes place. The resulting complex is however less catalytically active with respect PdCl_2_ or, more likely, less able to transform to the active species (Figure 21). 

### 5.2. ILs as precursors of carbene ligands

It is noteworthy that, despite the vast majority of the research in the area of ILs is focused on compounds derived from imidazole, due to the simplicity in preparing these derivatives and the generally lower melting points and viscosities as compared to other classes (such as ammonium-, phosphonium- or pyridinium-based ILs), at the beginning of the development of Heck protocols in ionic liquids they proved to be significantly less effective when compared to tetraalkylammonium salts in the absence of phosphine ligands [18,21]. However, it has been reported that the addition of a phosphine ligand when the reaction was carried out in [BMIM][PF_6_] resulted in a dramatic increase in the reaction rate (Table 3) [18]. 

It is also interesting to note that, under phosphine-free conditions, the reaction was not effective at 100 °C (entry 3), but when the temperature was raised to 140 °C the required cinnamate was obtained in 94% yield (entry 4). This result was attributed, once again, to the formation of Pd-NPs, which became active as catalyst at T > 100 °C [6].

Similarly, Herrmann and co-workers found that imidazole-based ILs gave less satisfactory results compared to tetraalkylammonium salts (Table 4) [21].

It has to be noticed that a particularly low GLC yield was observed when the noncoordinating [PF_6_] anion was used as counterion (entry 4). All these results clearly suggested that either different catalytically active species are involved in relation with the used ionic liquids, or that the reaction proceeds through different mechanistic pathways.

**Scheme 19:**
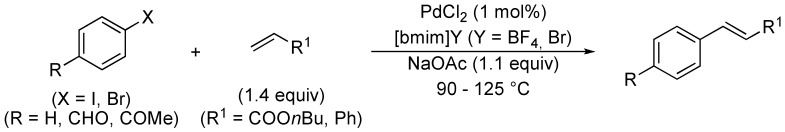
Heck reaction in imidazolium based ILs.

It is well established that the α-position of imidazolium salts can be deprotonated to form stabilized carbenes [76],and that these Arduengo-type carbene are employed as ligands in the area of transition metal-catalyzed reactions [77]. In 2000, as already stated, Xiao and co-workers obtained the first convincing evidence that carbene species are involved in Heck reactions carried out in imidazolium-based ionic liquids (Scheme 19) [29].

**Figure 22:**
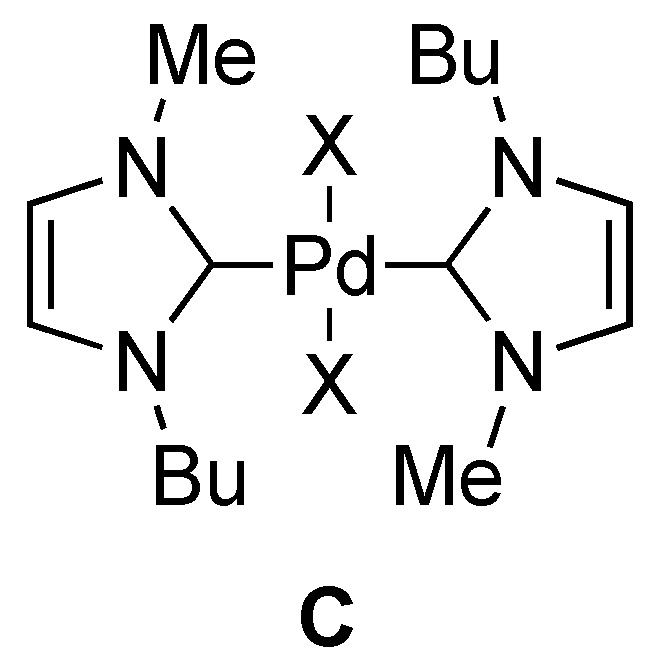
Isolated carbene palladium complex.

They found that the Heck reaction proceeded markedly more efficiently in the ionic liquid containing bromide as the counterion than in the analogous tetrafluoroborate salt. In conjunction with these observations, carbene complexes have been isolated by reaction of [bmim]Br with Pd(OAc)_2_, but non-carbene species were detected when [bmim][BF_4_] was used. The authors attributed the stability of the isolated carbene complex **C** (Figure 22) to the bromide ions (X = Br), while the presence of [BF_4_]^-^ probably converts **C** into an inactive complex [29].

Subsequent studies proved that the acidity of the α-position in imidazolium salts depends not only on the structure of the heterocyclic moiety, but also on the nature of the anionic partner [50,79]. These studies revealed that more basic anions (such as halides) resulted in imidazolium salts that undergo an easy deprotonation even in the absence of any added base (thus generating carbenes), while weakly coordinating anions (such as tetrafluoroborate or hexafluorophosphate anions) resulted in salts which necessitate strong external bases for the C-2 deprotonation [79]. As a consequence, it may be possible to conclude that imidazolium halides, or imidazolium-based ionic liquids containing coordinating (basic) anions, give rise to carbene Pd complexes which resulted stabilized by the same anions, while imidazolium salts containing non-coordinating anions furnished inactive Pd species, which can convert to Pd-NPs at elevated temperatures [73] or into active Pd catalysts in the presence of phosphine ligands [18].

These conclusions are in agreement with the negative result observed when a 2-methyl substituted glycerol-containing imidazolium salt, which cannot generate carbenes in the reaction medium, was used as the solvent in the Heck arylation of ethyl acrylate. In contrast, the analogous Cl or Br salts, that may be converted into Arduengo-type carbene ligands, resulted good solvents for the Heck reaction promoted by PdCl_2_ in the presence of NaOAc as the base [49].

Moreover, Lauth-de Viguerie and co-workers reported the use of palladate salts, generated from PdCl_2_ and [BMIM]Cl, as catalysts for the Heck reaction in [BMIM][PF_6_], which proved to be more effective than traditional precatalysts [80]. These results were attributed to the presence of NPs which served as reservoir of Pd species, while the formation of carbenes was excluded.

### 5.3. ILs and regioselectivity in the Heck reaction

**Scheme 20:**
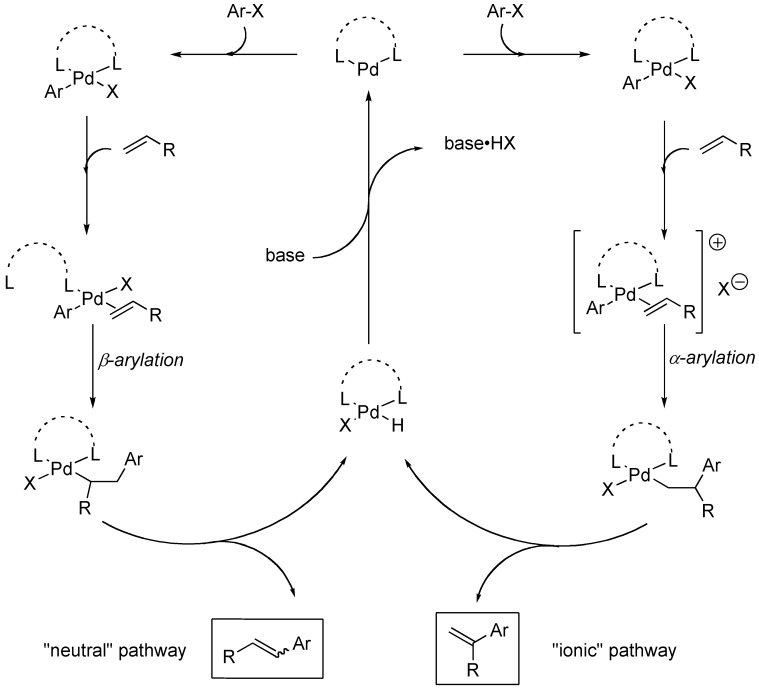
Proposed neutral and ionic pathways for the Heck reaction.

The regioselectivity of the Heck reaction is considered to be affected by the two generally accepted pathways: ionic versus neutral, as illustrated in Scheme 20 [81].

As depicted in Scheme 20, the neutral pathway, which is more sensitive to steric factors than to electronic factors, leads mainly to the formation of linear or β-substituted alkenes, while the ionic pathway, which depends on electronic factors than on steric factors, prevalently yields branched or α-products. The neutral pathway is characterized by dissociation of one of the coordinating neutral ligand L, while ionic pathway generates a vacant coordination site through the dissociation of the (pseudo)halide anion. As a consequence, with monodentate ligands and aryl halides the neutral pathway should dominate due to the easy dissociation of the ligand in comparison with the relatively strong Pd-X bond. In contrast, the liability of Pd-Y bond (where Y = OTf, OMs, OTs) means that the ionic route should be preferred when using low-coordinating anions, particularly in the presence of chelating bidentate ligands [31,83].

For reactions involving electron-deficient olefins (R = EWG) under “normal” Heck conditions, that imply the use of monodentate phosphines and aryl/vinyl halides, the products usually result from arylation/vinylation at the terminal β-position through the neutral pathway. However, under similar conditions the observed regioselectivity when electron-rich olefins (R = EDG) are reacted with halides might be poor, and a mixture of products is often obtained [11,83]. In contrast, the promotion of the ionic pathway by using bidentate ligands and pseudohalides such as triflates, tosylates or mesylates as labile counterions, as showed by Hallberg [11] and by Cabri [83], generated a highly reactive cationic palladium complex and resulted in an efficient regioselective α-arylation/vinylation of electron-rich olefins.

Unfortunately, problems such as poor availability, high cost and thermal liability limited the synthetic use of this approach, and now aryl/vinyl halides are used in the presence of a halide scavenger such as silver or thallium salts. However, the cost of silver and the toxicity of thallium severely prevent their use on a large scale.

**Scheme 21:**
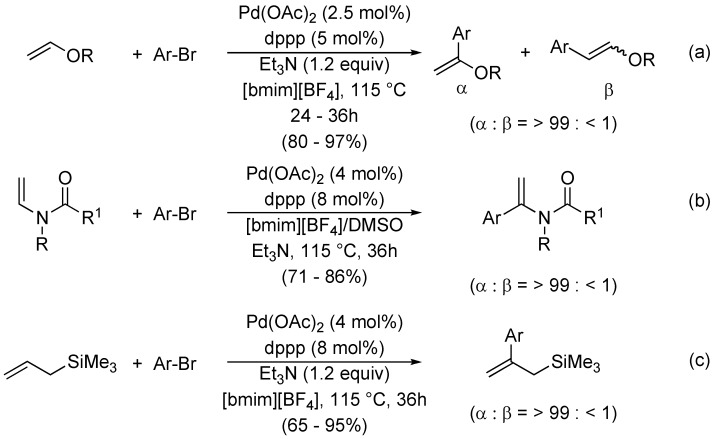
α-Arylated olefins from electron-rich olefins.

To overcome these problems, Xiao and co-workers reported the use of imidazolium-based ionic liquids in conjunction with Pd-dppp precatalyst for highly regioselective Heck arylations of electron-rich olefins [31,84,85]. As previously discussed, they supposed that the ionic pathway might be promoted by using ionic liquids as solvents, producing branched olefins without halide scavengers, according to the recent kinetic studies made by Amatore, Jutand and co-workers [86,87]. The strong electrostatic interactions existing in an ionic liquid would favour the generation of a Pd-olefin cation and a halide anion from two neutral precursors over the formation of a neutral Pd-olefin intermediate. In fact, they were able to obtain regioselectively and with high chemical yields α-arylated olefins starting from electron-rich olefins such as vinyl ethers, enamides, allyltrimethylsilane (Scheme 21) [31,84].

In addition, the group of Xiao observed that hydrogen-donating salts such as [HNEt_3_][BF_4_] could increase both the rate and the selectivity in ionic as well in molecular solvents, presumably by facilitating dissociation of bromide from Pd(II) complex (see eq.1) [85].

The selectivity of the Heck reaction is influenced not only by the reaction pathway, ionic or neutral, but also by other parameters including, among others, the choice of additives and the structure of the coupling partners.

**Scheme 22:**
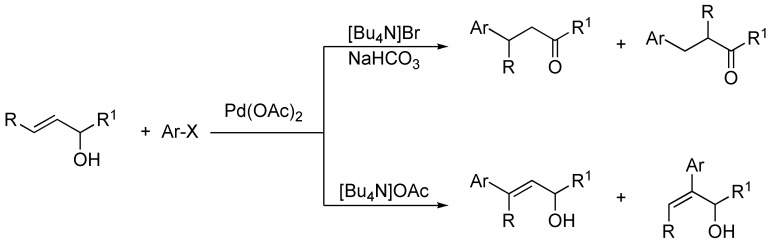
Heck arylation of allylic alcohols.

The influence of the base and the chosen ionic liquid on the selectivity was evidenced by Calò and co-workers [88]. In particular, they found that using [Bu_4_N][OAc] dually as solvent and base, the Heck arylation of allylic alcohols under phosphine-free conditions resulted highly selective towards the formation of aromatic conjugated alcohols, while with [Bu_4_N]Br as solvent and NaHCO_3_ as the base the aromatic carbonyl compounds were selectively obtained (Scheme 22) [88].

**Scheme 23:**
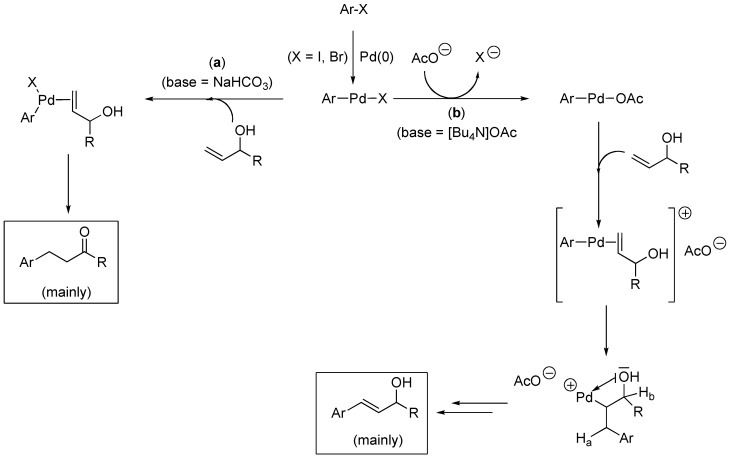
Possible reaction pathways.

The authors, in order to explain the obtained results, supposed that the reaction can follow two different pathways, depending on the nature of the ligand (X) bonded to palladium before the migratory insertion of the aryl group on the olefinic double bond (Scheme 23) [88]. 

In particular, when [Bu_4_N]Br is used as solvent, and a conventional base such as NaHCO_3_ is employed, ligand X will be bromide or iodide anion (path a) and the reaction follows the neutral pathway (path a). For steric reasons, the migratory insertion of the aryl group predominantly occurs to the β-position of the alkenol, giving rise to β-arylated carbonyl products.

In contrast, when [Bu_4_N][OAc] is used as the reaction medium, an anionic metathesis probably occurs, and the acetate anion readily dissociates to give a cationic complex. This may explain the higher catalytic activity displayed by Pd(OAc)_2_ in [Bu_4_N][OAc], which allows the metal to activate iodo- and bromoarenes at low reaction temperatures (r.t. and 60 °C, respectively, in contrast with the 100 °C and 130 °C required in [Bu_4_N]Br). In this case, the migratory insertion on the double bond occurs prevalently on the β-position due to the formation of a chelate structure, which impedes also the hydrogen atom (H_B_) to adopt the *syn*-relationship with Pd, necessary for the β-elimination. Hence, the abstraction of the benzylic hydrogen atom (H_A_) remains the only possible pathway.

## 6. Conclusions

The research of more sustainable procedures prompted organic chemists to look for more eco-compatible and more economical transition metal-catalyzed reactions. In the last ten years, a growing number of palladium catalyzed Heck reactions in ILs have been proposed as greener alternative to the classical cross-coupling procedure. Homogeneous and heterogeneous conditions have been explored showing that ILs are not only suitable solvents for the Heck reaction, but their unique physico-chemical properties are able to change the course of the reaction, activating and/or stabilizing intermediates or transition states in the reaction mechanisms. Consequently, Heck reactions performed in ILs, or ILs containing environments, may have higher reaction rates, and may be characterized by a higher control on the regio- and stereoselectivity of the coupling products. The involvement of carbene complex, palladium nanoparticles and palladium anionic complexes has been evidenced depending on ILs structure and reaction conditions. In spite of the numerous interesting reports mentioned in this review, the study of the Heck reaction in ILs is still strongly related to the development of the IL; only simple reactions such as the coupling of aryl halides with cinnamates or styrene have been investigated. Thus, in the future, the use of ILs in Heck reactions involving more complicated substrates could give important indications about the possibility of application of these alternative media on large scale reactions.

## Figures and Tables

**Figure 13:**
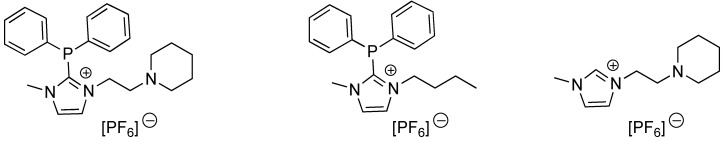
Mixture of monofunctionalized ILs.

**Table 1:**
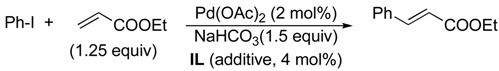
Influence of the counteranion on the efficiency of the Heck reaction.

IL	Additive	Reaction temp. (°C)	Reaction time (h)	Yield (%)
[C_6_Py][PF_6_]	-	80	72	42
[C_6_Py][BF_4_]		80	72	99
[C_6_Py]Cl	Ph_3_P	40	24	82
[C_6_Py]Cl		100	24	99

**Table 2:**
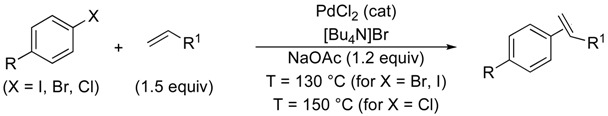
Ligand-free Heck arylation in [Bu_4_N]Br. [21]

Entry	R	R^1^	X	PdCl_2_ (mol%)	t (h)	Yield GLC (%)^(a)^	TON
1	Me	Ph	I	0.01	14	100	10000
2	COMe	O-*n*-Bu	Br	0.2	14	98	490^(b)^
3	H	4-MeOC_6_H_4_	Br	0.1	14	50	500
4	H	4-MeC_6_H_4_	Br	0.1	14	68	680
5	H	4-CF_3_C_6_H_4_	Br	1.0	14	91	910
6	H	O-*n*-Bu	Br	0.2	14	69	345^(c)^
7	H	*n*-butylmethacrylate	Br	1.0	14	100	100
8	OMe	O-*n*-Bu	Br	0.2	13	52	260^(d)^
9	OMe	Ph	Br	0.1	14	69	690
10	NO_2_	Ph	Cl	0.1	19	100	1000
11	COMe	Ph	Cl	0.1	14	97	970
12	H	Ph	Cl	5	45	89	18
13	OMe	Ph	Cl	3	40	5	2

(a) The yield is referred to the (*E*). (b) 42% (*E*), 37% (*Z*), 20% (1/1). (c) 17% (*E*), 24% (*Z*), 29% (1/1). (d) 10% (*E*), 14% (*Z*), 27% (1/1).

**Table 3:**
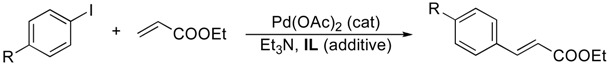
Heck reaction in imidazole-based ILs.

Entry	IL	Additive	Reaction temp. (°C)	Reaction time (h)	Yield (%)
1	[C_6_Py]Cl	-	40	24	99
2	[pmim]Cl	-	80	72	10
3	[bmim][PF_6_]	-	100	20	7
4	[bmim][PF_6_]	-	140	18	94
5	[bmim][PF_6_]	Ph_3_P	100	1	95-99

**Table 4:**
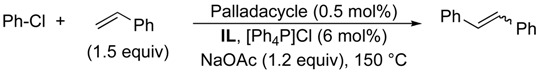
Palladacycle-mediate Heck reaction in ILs.

Entry	IL	Reaction time (h)	GLC yield (%)
1	[Bu_4_N]Br	18	51
2	[pmim]Br	19	22
3	[bpim]Br	16	11
4	[bbim]PF_6_	15	5
